# Study on the association and phase separation behavior of surfactants and promethazine hydrochloride: impact of ammonium electrolytes

**DOI:** 10.1039/d3ra07493e

**Published:** 2024-02-15

**Authors:** Afzal Hossain Shah, S. M. Rafiul Islam, Munirah D. Albaqami, Tajmul Hasan, Dileep Kumar, Saikh Mohammad Wabaidur, Mohd Zahid Ansari, Md. Anamul Hoque, D. M. Shafiqul Islam, Mahbub Kabir

**Affiliations:** a Department of Chemistry, Jahangirnagar University Savar Dhaka 1342 Bangladesh; b Department of Chemistry, College of Science, King Saud University Riyadh 11451 Saudi Arabia; c Laboratory for Chemical Computation and Modeling, Institute for Computational Science and Artificial Intelligence, Van Lang University Ho Chi Minh City Vietnam kumar.dileep@vlu.edu.vn +84 943720085; d Faculty of Applied Technology, School of Technology, Van Lang University Ho Chi Minh City Vietnam; e School of Materials Science and Engineering, Yeungnam University Gyeongsan 712749 South Korea

## Abstract

In the current study, the association and phase separation of cationic tetradecyltrimethylammonium bromide (TTAB) and nonionic Triton X-100 (TX-100) surfactants with promethazine hydrochloride (PMH) were investigated in aqueous ammonium-based solutions. The micellization nature of the TTAB and PMH drug mixture was examined by evaluating critical micelle concentration (CMC) and counterion binding extent (*β*) at different salt contents and temperatures (298.15–323.15 K). Micelle formation in the TTAB + PMH mixture was enhanced in the presence of ammonium salts, whereas the process was delayed with an increase in temperature in the respective salt solution. With an increase in salt content, the cloud point (CP) of the TX-100 + PMH mixture decreased, which revealed that the respective progression occurred through the salting out phenomenon. In micellization and clouding processes, the changes in free energies Δ*G*^0^_m_ and Δ*G*^0^_c_ were found to be negative and positive, respectively, demonstrating that the corresponding processes are spontaneous and non-spontaneous. Standard enthalpies (Δ*H*^0^_m_/Δ*H*^0^_c_) and standard entropies (Δ*S*^0^_m_/Δ*S*^0^_c_) for the association and clouding processes, respectively, were also calculated and discussed. The core forces amid TTAB/TX-100 and PMH in the manifestation of electrolytes are dipole–dipole and hydrophobic forces among the employed components according to the values for Δ*H*^0^_m_/Δ*H*^0^_c_ and Δ*S*^0^_m_/Δ*S*^0^_c_, respectively.

## Abstract 2

Representative plot of CMC of the TTAB + PMH (3 mmol kg^−1^) system *vs.* ammonium salt contents at 303.15 K.

## Graphical abstract

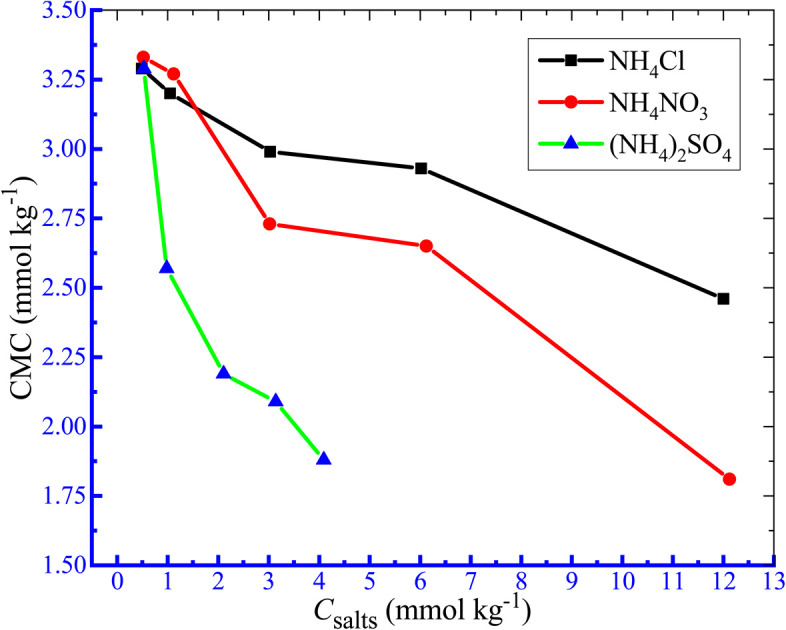

## 1. Introduction

Numerous amphiphilic compounds have been extensively used in a variety of industries, including farming, food engineering, oil industry, environmental and biological areas, pharmaceutical technology, textiles, and metallurgy.^1–10^ These molecules are composed of two parts: (i) a hydrophobic component that prefers oil to water and (ii) a hydrophilic component that prefers water to oil. Above their CMC, these amphiphilic compounds in an aqueous environment can form a molecular self-assembly, which is referred to as a micelle.^11–18^ Natural and spontaneous processes, such as self-assembly and self-organization, mostly depend on non-covalent interactions (van der Waals, H-bonding, hydrophobic/hydrophilic, and electrostatic).^19^ The potential of micelle solutions as useful molecular assemblies for applications in various pure and practical sciences has nowadays gained much attention from researchers. Micelle solutions can serve as models for several biochemical and pharmacological systems. As a result, the hydrophobic cores of surfactant aggregates preserve their ability to solubilize water-insoluble compounds.^20^

**Scheme 1:**
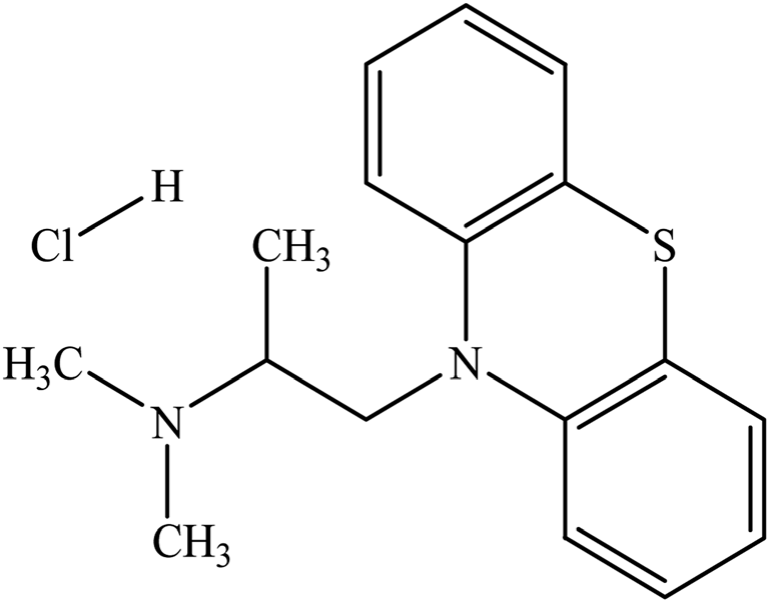
Molecular structure of PMH.

**Scheme 2:**
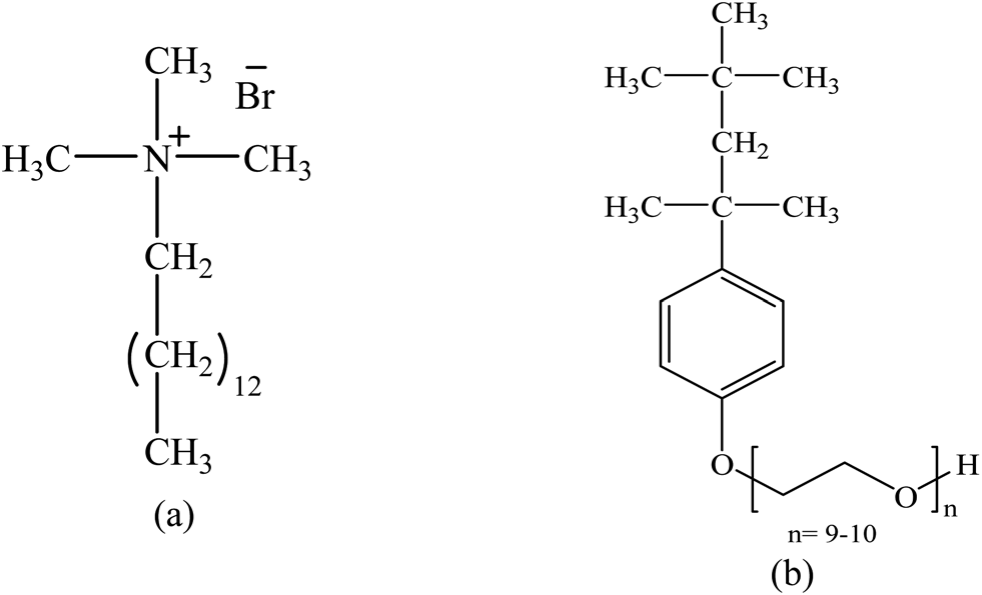
Molecular structures of (a) TTAB and (b) TX-100.

In this study, we plan to evaluate the solution properties, association nature, and physico-chemical variables of the mixture of promethazine hydrochloride (PMH) (Scheme 1) and tetradecyltrimethylammonium bromide (TTAB)/nonionic Triton X-100 (TX-100) (Scheme 2) in different ammonium salt media (NH_4_Cl, NH_4_NO_3_, (NH_4_)_2_SO_4_, (NH_4_)_2_HPO_4_, and (NH_4_)_2_SO_4_·FeSO_4_·6H_2_O, (AFS; ammonium ferrous sulfate)). AFS has been used as a food preservative in analytical chemistry as a substitute for ferrous sulphate. The modification of metabolites such as NH_3_, glucose (C_6_H_12_O_6_), lactate (C_3_H_5_O_3_^−^), and glycine (C_2_H_5_NO_2_) in the human body is directly influenced by liver diseases.^21^ The ammonium ion (NH_4_^+^) is generated when ammonia and hydrogen ions combine and are expelled through urine, helping to maintain the normal pH of our bodies. Because the production of NH_4_^+^ plays a noteworthy role in regulating the pH of the human body, the contents of NH_3_ change during various diseases, such as liver diseases.^22^ PMH is a phenothiazine drug used to treat allergies, nausea, and vomiting-related illnesses. Additionally, it is used as a sedative before and after surgery and to stop a runny nose from a common cold. The present studies of TTAB/TX-100 + PMH in ammonium salt media might provide important knowledge that will be beneficial for the progress of drug delivery systems and drug formulations.

The phenomena of clouding are well known and have been observed in nonionic surfactants; as temperature increases, the system turns cloudy and phases out at a specific temperature (CP).^23–30^ The cloud point depends on the change in additives.^31–33^ Among them, TX-100 is a fascinating non-ionic surfactant in which an aromatic ring is present in the middle of the structure acting as a bridge between lyophobic polyethylene chains and a lyophilic portion.^22^ The solubility behavior and pattern of TX-100 are quite amazing. The TX-100 surfactant compound has several notable uses in the production of biopharmaceuticals, including the infiltration of cell membranes and the deactivation of lipid-encased viruses. TX-100 is used as an ingredient of influenza vaccines, a constituent of the lysis buffer for DNA extraction and a species for reducing the surface tension of water throughout immunostaining.^34–37^

Although many works have been reported on the cloudy generation and micellization of surfactants in the manifestation of drugs,^38–45^ the behavior of the respective processes in the presence of ammonium-based salts is still unknown. Rub *et al.*^42^ reported the phase partition and assembly of TX-100/TTAB + metformin hydrochloride drug solutions in different hydrotropic media. Ahsan *et al.*^23^ reported the nature of micelle generation and cloudy formation of a sodium dodecyl sulfate (SDS)/TX-100 and lomefloxacin hydrochloride drug mixture. They determined and explored the thermodynamic parameters and interaction forces among them. Recently, we studied the interaction of TTAB/TX-100 with PMH in potassium salts/alcoholic additives.^44,45^ Herein, we explore the impacts of (i) concentrations of ammonium-based electrolytes on CMC and CP of a working system, (ii) temperature on TTAB + PMH micellization in ammonium salt system; (iii) thermodynamic variables (free energy (Δ*G*^0^_m_/Δ*G*^0^_c_), enthalpy (Δ*H*^0^_m_/Δ*H*^0^_c_) and entropy (Δ*S*^0^_m_/Δ*S*^0^_c_)), and (iv) enthalpy–entropy compensation variables of micellization/clouding processes in the attendance of employed salts.

## 2. Experimental

### 2.1. Materials

The procured materials in the working processes were of reagent grades, and these have been applied without any purification. The origin and purity of the materials used are shown in Table 1.

**Table 1:** Source and purity of the employed materials used in the experiments

Chemical	Source	Mass fraction purity	CAS number
TTAB	Sigma-Aldrich, USA	0.99	1119-97-7
TX-100	Daejung Chemicals & Metals Co. Ltd., Gyeonggi, Korea	—	9002-93-1
PMH	Sigma-Aldrich, India	0.98	58-33-3
NH_4_Cl	Merck, Mumbai, India	≥0.99	12125-02-9
NH_4_NO_3_	Merck, Mumbai, India	≥0.98	6484-52-2
(NH_4_)_2_SO_4_	Merck, Mumbai, India	≥0.99	7783-20-2
(NH_4_)_2_HPO_4_	Scharlau, Spain	≥0.98	7783-28-0
AFS	BDH Chemicals Ltd., England	≥0.99	7783-85-9
H_2_O	Distilled de-ionized		

### 2.2. Approach of conductivity

The conductivity measurements were carried out by applying a conductivity meter (Jenway 4510, UK) fitted with a dip cell (cell constant = 0.97 cm^−1^) at various compositions and temperatures (298.15–323.15 K). TTAB (50 mmol kg^−1^) stock solutions with a predetermined concentration of additives (H_2_O + PMH (3 mmol kg^−1^) + NH_4_Cl/NH_4_NO_3_/(NH_4_)_2_SO_4_) were made. The conductivity (*κ*) was assessed by adding concentrated solutions one at a time into either a set concentration of additive (H_2_O + PMH (3 mmol kg^−1^) + NH_4_Cl/NH_4_NO_3_/(NH_4_)_2_SO_4_) solutions based on the literature.^46–48^ The desired temperature of the solutions was sustained by utilizing a thermostated water bath (JSRC-13C, Korea). The micelle development concentration (CMC) is determined from the break point acquired in the *κ vs.* [surfactant] plots.^49–52^

### 2.3. Assessment of cloud point (CP)

The solutions containing TX-100 and PMH were formulated in the desired solvent (aq. salt solutions with specific concentrations). In a carefully regulated heating and cooling experiment, the cloud points of solutions were measured visually using the described Albertsson method,^53^ which was also adapted by Blankschtein *et al.*^54^ Solutions were placed in a thin Pyrex glass tube, which was then sealed and heated gradually in a water bath with internal circulation and a digital temperature display that had an accuracy of 0.1 K. Near the CP, the temperature increased gradually at a rate of 0.5 K min^−1^ while being continuously stirred. Visual observation was made of the clouding site at the beginning of the phenomenon. The system was then given time to cool when the heater was turned off. Once more, the temperature was recorded at turbidity clearance. The CP of the system was determined to be the mean of the two temperatures. To determine the right CP values, the procedure was carried out at least 3 times, with the mean of the concurrent run acting as the ultimate CP. The literature^55,56^ provides a detailed description of the experimental process.

## 3. Results and discussion

### 3.1. Conductivity measurements

#### 3.1.1. CMC and *β* of a drug-surfactant system in aq. ammonium salts media

**Fig. 1:**
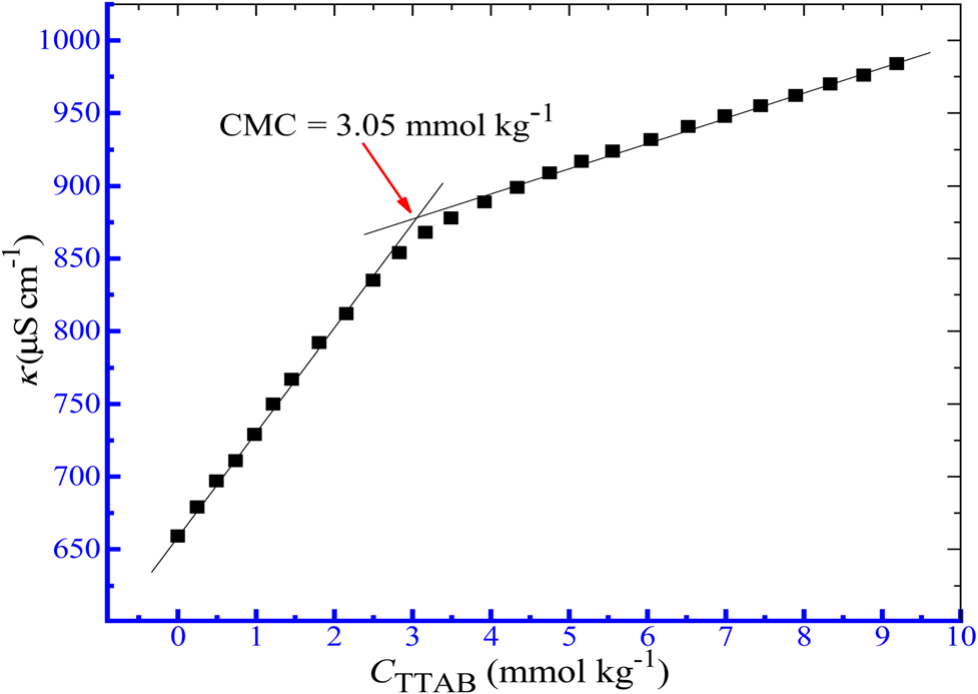
Graph of *κ vs.* [TTAB] for the association of the TTAB + PMH system in aq. NH_4_Cl (3.031 mmol kg^−1^) environment at 303.15 K.

The aggregation of TTAB in the presence of additives is explored in the current investigation from the changes in the conductivity of ionic surfactants in the solution phase. The aggregate formation of surfactants has been reported to rely on the presence of additives and experimental conditions.^50–52^ The hydrophilic and hydrophobic characteristics of the surfactant moiety, the presence of an aqueous additive solution, and environmental factors, such as temperature, affect the CMC value. When the energy produced by the association of the hydrophobic portion of the monomer is adequate to overcome electrostatic repulsion among ionic head groups, the entropy loss that accompanies the aggregation, micellization occurs.^57^ Herein, the conductivity technique is employed to assess the CMC of the TTAB + PMH system in the presence of ammonium salt media. Three ammonium salts, such as NH_4_Cl, NH_4_NO_3_ and (NH_4_)_2_SO_4_, were used in the current investigation. Fig. 1 demonstrates the change in *κ* with the augmentation of surfactant concentration for the TTAB + PMH mixed solution in aq. ammonium chloride environment. Only a CMC value was observed for the investigated surfactant content for the TTAB + PMH system in the presence of ammonium salts.

In our previous work,^58^ the effect of PMH on the assembly of TTAB in an aqueous medium where the addition of the drug favoured the micelle formation of TTAB (the CMC values decreased as a function of PMH concentrations). In that investigation, the used concentrations of PMH were 1, 3, 5, 7 and 10 mmol kg^−1^ in an aqueous medium, whereas the effect of temperature and addition of K-based salts on the aggregation of the TTAB + PMH mixture was performed considering a 3 mmol per kg PMH drug. The CMC value of 3.71 mmol kg^−1^ for the assembly of the TTAB + PMH mixture in a water medium was obtained at 310.55 K.^58^ The addition of KCl and K_2_SO_4_ further facilitated the aggregation of the TTAB + PMH mixture.^58^ Additionally, doctors prescribe 6.5 mg to 25 mg PMH drug daily, which may vary with the patient's condition. Again, this drug undergoes further dilution when it is administered in the body. Consequently, considering these facts, in the present investigation, 3 mmol per kg PMH drug was kept fixed in the entire examination of the micellization of the TTAB + PMH mixture in salt media. The addition of ammonium electrolytes to the solution might affect CMC. This affects the amount of counterion binding, which in turn affects the attraction of the ionic head groups to one another and changes the CMC. The following equation^59,60^ is used to empirically quantify this effect:

1 Log CMC = −*a* log *C*_t_ + *b*,

where *a* and *b* are two constants for a specific ionic head group and *C*_t_ represents the entire counterion concentration. The extent of micellization ionization (*α*) was measured using the following equation:2
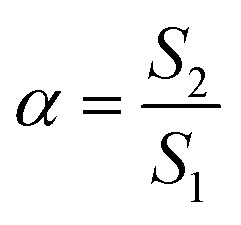
where *S*_1_ and *S*_2_ denote the extent of the slope value of the linear line below and above CMC in the *κ vs.* [TTAB] plot, respectively. The degree of counterion bounds to micelles (*β*) can be obtained by the subtraction of *α* from the unity.^61^ The effect of three ammonium salts, such as NH_4_Cl, NH_4_NO_3_ and (NH_4_)_2_SO_4_, on the variables associated with the aggregation of surfactant-drug systems was examined in the current investigation. The concentrations of NH_4_Cl, NH_4_NO_3_ and (NH_4_)_2_SO_4_ were chosen in the range of 0.4911–12.00 mmol kg^−1^, 0.5221–12.12 mmol kg^−1^ and 0.5333–4.097 mmol kg^−1^, respectively, while the values of ionic strength (*I*) of respective ammonium salts are provided in Table 2. Fig. 1 demonstrates the change in *κ* with the augmentation of surfactant concentration for the TTAB + PMH mixed solution in aq. ammonium chloride environment.

**Table 2:** CMC and *β* values of the TTAB + PMH (3 mmol kg^−1^) system in different ammonium salt solutions at 303.15 K

Medium	*C*_salts_, mmol kg^−1^	*I*_salts_, mmol kg^−1^	CMC, mmol kg^−1^	*X*_CMC_ (×10^5^)	*β*
H_2_O + NH_4_Cl	0.4911	0.4911	3.29	5.928	0.76
	1.050	1.050	3.20	5.766	0.73
	3.031	3.031	3.05	5.495	0.73
	6.022	6.022	2.93	5.279	0.72
	12.00	12.00	2.46	4.432	0.73
H_2_O + NH_4_NO_3_	0.5221	0.5221	3.33	6.000	0.78
	1.124	1.124	3.27	5.892	0.77
	3.024	3.023	2.73	4.919	0.76
	6.125	6.125	2.65	4.774	0.66
	12.12	12.12	1.81	3.261	0.76
H_2_O + (NH_4_)_2_SO_4_	0.5333	1.600	3.29	5.928	0.66
	1.014	2.942	2.57	4.631	0.78
	2.113	6.339	2.19	3.946	0.79
	3.143	9.429	2.09	3.766	0.77
	4.097	12.29	1.88	3.387	0.79

**Fig. 2:**
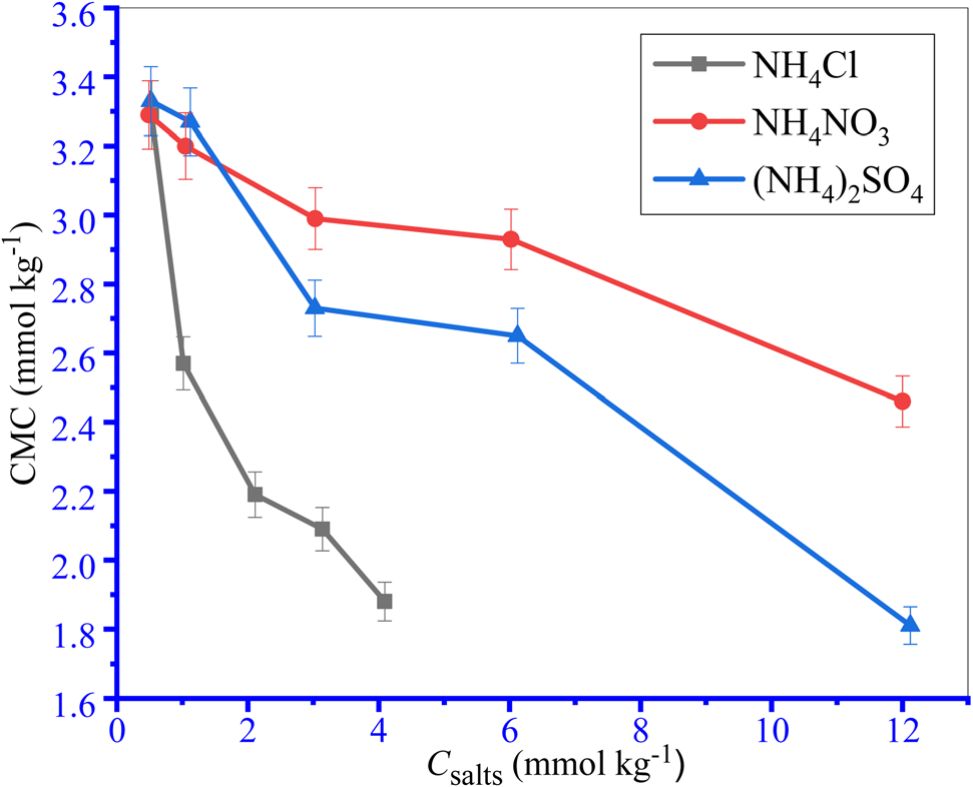
The plots of CMC of the TTAB + PMH (3 mmol kg^−1^) system *vs.* ammonium salt contents at 303.15 K temperature. Error in CMC is ±3.0%.

Only a CMC value was observed for the investigated surfactant content for the TTAB + PMH system in the presence of ammonium salts. The values of CMC and mole fractional values of CMC (*X*_CMC_) along with counter ion binding (*β*) of TTAB + PMH system in the incidence of ammonium salts are shown in Table 2 and Fig. 2. The CMC values of the TTAB + PMH system experience a reduction in the presence of ammonium salts, and the reducing trend also undergoes augmentation with the enhancing concentration of salts. The CMC of the TTAB + PMH system at 303.15 K in aq. ammonium salts demonstrates the following order: CMC (NH_4_Cl) > CMC (NH_4_NO_3_) > CMC ((NH_4_)_2_SO_4_). The shrinking of the thickness along with electric double-layer potential happens in the manifestation of salts, which reduces the repulsion amongst the charged head groups, thereby generating a favorable environment for the aggregation of surfactant molecules. The (NH_4_)_2_SO_4_ imparts a superior effect to NH_4_Cl and NH_4_NO_3_ to produce a satisfactory situation for the assembly. The sulfate ion (SO_4_^2−^) is a robust kosmotropic ion with a greater charge density related to the chloride and nitrate ions because these CMC values are lesser than chloride and nitrate ions.^44,62,63^

However, the chloride ion is deliberated as a chaotropic identity with a low charge density and has poor power that disrupts water arrangement; thus, the extent of the association of amphiphilic monomers is reduced. We consider a range of concentrations of salts containing chloride, nitrate and sulfate ions containing salts. If the concentration of chloride/nitrate ions and sulfate ions doubles, the CMC values still show a decreasing trend. When 3.031 M NH_4_Cl and 0.9808 M (NH_4_)_2_SO_4_ solutions (both solutions have the same ionic strength (*I*) of 3 mmol kg^−1^) are used, their CMC values are 3.05 and 2.57 mmol kg^−1^ respectively. However, when 6.022 M NH_4_Cl solution is used, the CMC is 2.93 mmol kg^−1^, which is comparatively higher than the CMC for the (NH_4_)_2_SO_4_ solution of identical ionic strength.

#### 3.1.2. Outcome of the temperature variation on the aggregate formation by the TTAB + PMH system in aq. ammonium salt media

**Fig. 3:**
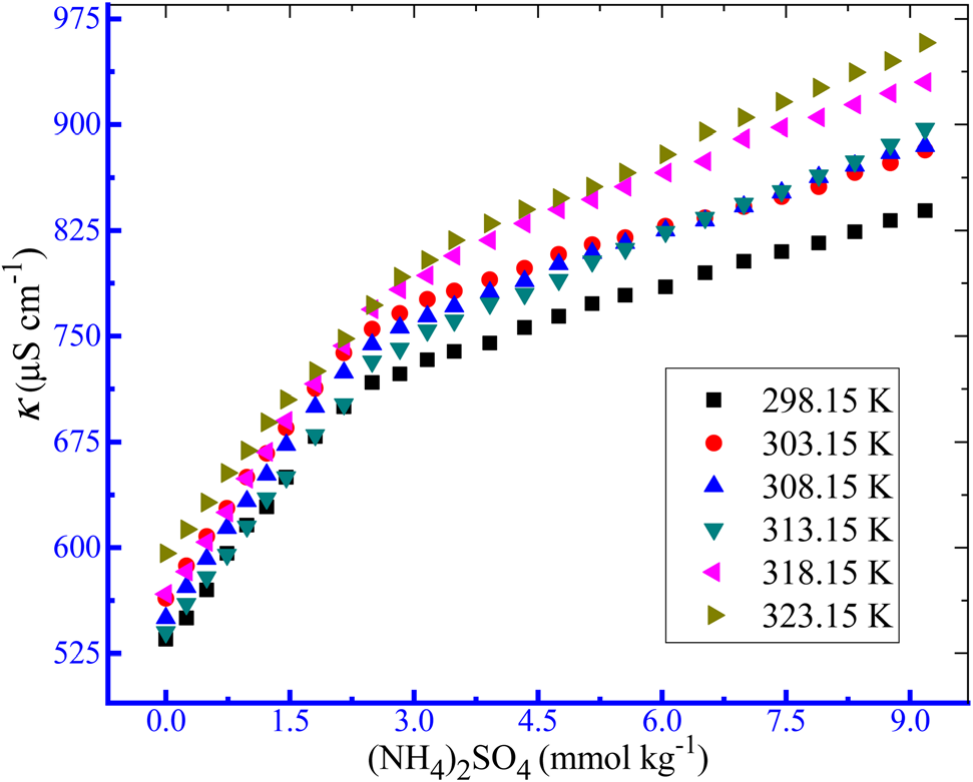
Variations in *κ* with an increase in [TTAB] for the assembly of the employed ionic amphiphile + PMH (3.0 mmol kg^−1^) mixture in aq. (NH_4_)_2_SO_4_ media.

**Fig. 4:**
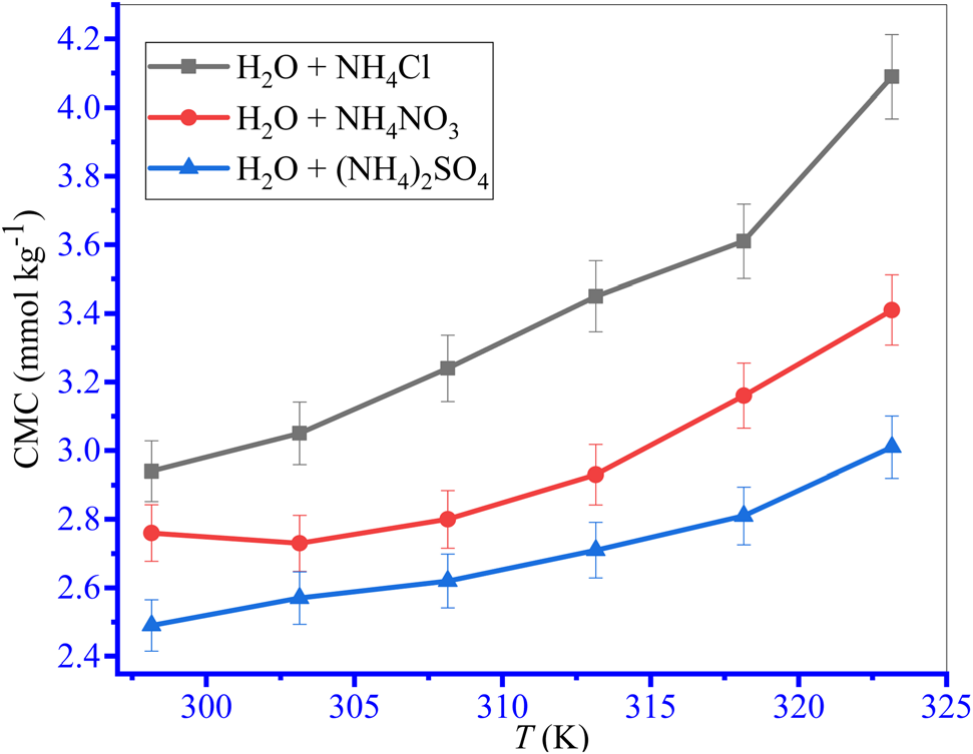
The effect of temperature on the aggregation of TTAB (50 mmol kg^−1^) + PMH (3 mmol kg^−1^) systems in ammonium electrolyte media. The error in CMC is ±3.0%.

The changes in conductivities with the growing contents of TTAB in the circumstance of the TTAB and PMH mixed solution in aq. system of NH_4_Cl, NH_4_NO_3_ and (NH_4_)_2_SO_4_ at study temperatures are discussed in the current investigation. Herein, to inspect the influences of temperature on the aggregation of TTAB + PMH under identical experimental conditions, fixed concentrations of NH_4_Cl, NH_4_NO_3_ and (NH_4_)_2_SO_4_ were 3, 3 and 1 mmol kg^−1^, respectively. These concentrations were chosen to maintain the identical ionic strength of the three salts used. Fig. 3 depicts the fluctuations of *κ* with the increase in [TTAB] for the aggregation of the TTAB + PMH (3.015 mmol kg^−1^) system in aq. (NH_4_)_2_SO_4_ solutions. The nature of change in CMC with the growing temperature for TTAB + PMH micelles in the occurrence of NH_4_Cl, NH_4_NO_3_ and (NH_4_)_2_SO_4_ at different temperatures (298.15–323.15 K) is illustrated in Fig. 4. The mole fractional values of CMC and *β* values obtained from the current investigation at working temperature are presented in Table 3. The CMC values have the propensity to be enhanced with the intensification of temperature in the presence of NH_4_Cl, NH_4_NO_3_ and (NH_4_)_2_SO_4_. The upsurge in CMC for TTAB + PMH micelles in aq. ammonium salt solutions suggests that the micelle formation of TTAB is delayed with the increase in experimental temperatures. Consequently, the aggregation of the working system occurs with the participation of a larger extent of TTAB as the study temperature increases in the employed salt solution. It is imperative to remark that the CMC values demonstrate almost a plateau in the CMC *vs. T* plot up to 303.15 K. Later, the CMC values have a habit of growth with an increase in temperatures, and the outcome is acquired in the case of aq. NH_4_NO_3_ solution. Two factors significantly affect changes in CMC values through changes in temperature. The types of hydrations surrounding monomeric amphiphile surfactant molecules and PMH-facilitated TTAB micelles alter as the temperature increases. The aggregated TTAB system exclusively experiences hydrophilic hydration, while the monomeric amphiphile system experiences both hydrophobic (H_2_O arrangement surrounding the non-polar moieties) and hydrophilic (H_2_O organization close to the polar portions) hydrations. As the temperature increases, both groups of hydrations tend to diminish.^64^ The micelle formation is favored owing to the fall of hydrophilic hydration (H_2_O structure near the polar heads), while this process is hampered by a decrease in hydrophobic hydration (H_2_O structure neighboring the nonpolar segments).^65–68^ Thus, the first factor dominates the second one with the increase in temperature in almost all three ammonium salt solutions used herein in the current study.

**Table 3:** Different physical parameters for the assembly of amphiphile + drug mixture in aq. ammonium salt media at investigational temperatures

Medium	*C*_salts_, mmol kg^−1^	*T*, K	CMC, mmol kg^−1^	*X*_CMC_ (×10^5^)	*β*
H_2_O + NH_4_Cl	3.011	298.15	2.94	5.297	0.79
	3.011	303.15	3.05	5.495	0.73
	3.011	308.15	3.24	5.838	0.75
	3.011	313.15	3.45	6.216	0.65
	3.011	318.15	3.61	6.504	0.71
	3.011	323.15	4.09	7.369	0.64
H_2_O + NH_4_NO_3_	3.024	298.15	2.76	4.973	0.77
	3.024	303.15	2.73	4.919	0.76
	3.024	308.15	2.80	5.045	0.76
	3.024	313.15	2.93	5.279	0.73
	3.024	318.15	3.16	5.693	0.69
	3.024	323.15	3.41	6.144	0.68
H_2_O + (NH_4_)_2_SO_4_	1.014	298.15	2.49	4.486	0.77
	1.014	303.15	2.57	4.631	0.78
	1.014	308.15	2.62	4.721	0.74
	1.014	313.15	2.71	4.883	0.69
	1.014	318.15	2.81	5.063	0.73
	1.014	323.15	3.01	5.423	0.64

#### 3.1.3. Thermodynamics of micellization

The study of thermodynamics specifies as a support to identify the nature of the relationship among the used components. The thermodynamic parameters of the TTAB + PMH mixed systems in the presence of ammonium salts are evaluated by employing eqn (3)–(7):^69,70^

3 Δ*G*^0^_m_ = (1 + *β*)*RT* ln *X*_CMC_,

4
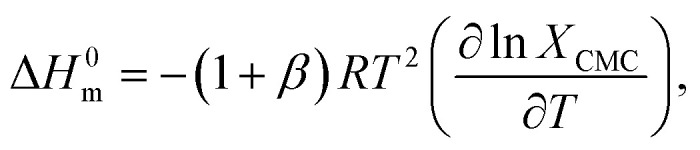

**Fig. 5:**
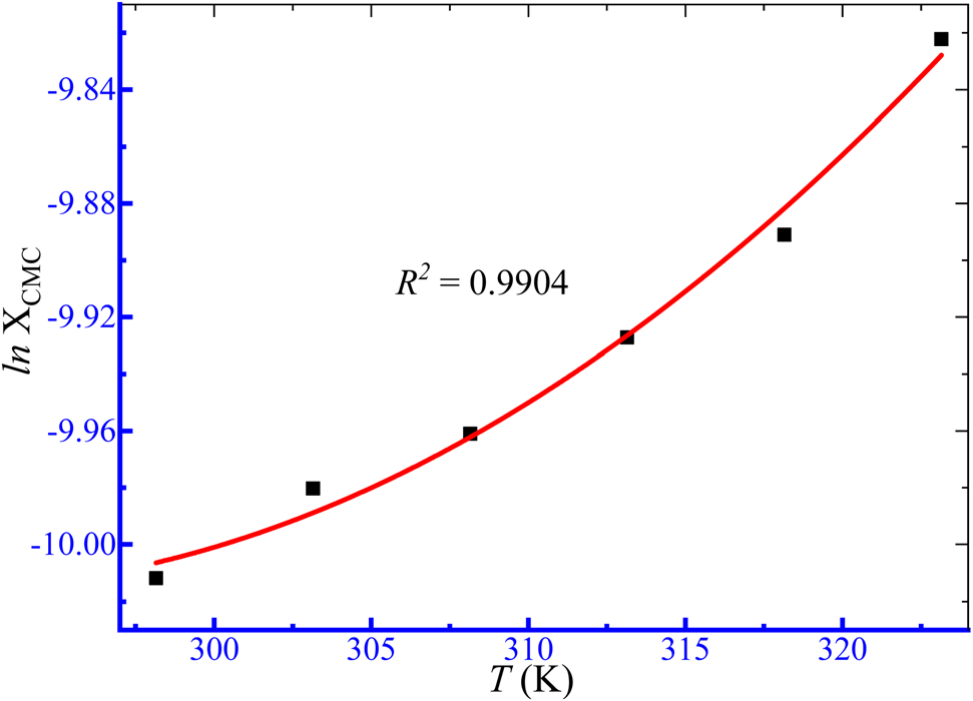
Ln *X*_CMC_*versus T* plot for micellization of TTAB + PMH (3 mmol kg^−1^) in an aq. (NH_4_)_2_SO_4_ solution.

5 ln *X*_CMC_ = *A* + *BT* + *CT*^2^,

6 Δ*H*^0^_m_ = −(1 + *β*)*RT*^2^[*B* + 2*CT*],

7
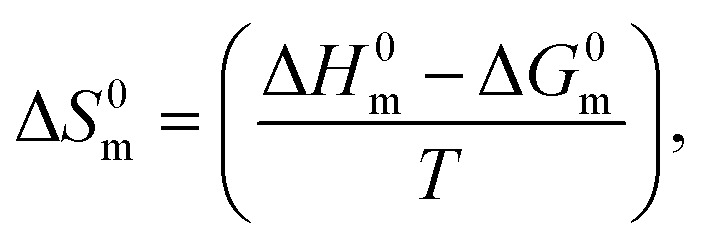
where *X*_CMC_ represents the mole fractional CMC values. The symbols *A*, *B* and *C* are regression constants obtained by the polynomial fitting (order 2) of the ln *X*_CMC_*vs. T* plots (Fig. 5), as shown in Table 4.

**Table 4:** Regression constants (*A*, *B*, and *C*) values for TTAB + PMH (3 mmol kg^−1^) aggregation in the aq. solution of ammonium-based salts

Medium	*C*_salts_, mmol kg^−1^	*A*	*B*	*C*	*R* ^2^
H_2_O + NH_4_Cl	3.011	7.441	−0.1237	0.0002	0.9743
H_2_O + NH_4_NO_3_	3.024	51.525	−0.4047	0.0007	0.9254
H_2_O + (NH_4_)_2_SO_4_	1.014	5.3111	−0.1054	0.0002	0.9904

In aq. ammonium salts, the Δ*G*^0^_m_ values of the TTAB + PMH association are acquired to be negative (Table 5). The negative values of Δ*G*^0^_m_ for TTAB + PMH association in ammonium salts within the working temperature range demonstrate the following order: −Δ*G*^0^_m_ (aq. (NH_4_)_2_SO_4_) > −Δ*G*^0^_m_ (aq. NH_4_NO_3_) > −Δ*G*^0^_m_ (aq. NH_4_Cl). Table 5 also illustrates that at fixed salt content the −Δ*G*^0^_m_ values are augmented with the growing study temperature.

**Table 5:** Values of Δ*G*^0^_m_, Δ*H*^0^_m_, and Δ*S*^0^_m_ for the studied system in aq. ammonium salts at different temperatures

Medium	*C*_salts_, mmol kg^−1^	*T*, K	Δ*G*^0^_m_, kJ mol^−1^	Δ*H*^0^_m_, kJ mol^−1^	Δ*S*^0^_m_, J mol^−1^ K^−1^
H_2_O + NH_4_Cl	3.011	298.15	−24.41	5.860	101.5
	3.011	303.15	−24.77	3.231	92.38
	3.011	308.15	−24.98	0.6114	83.03
	3.011	313.15	−25.16	−2.102	73.63
	3.011	318.15	−25.50	−5.117	64.09
	3.011	323.15	−25.57	−7.934	54.59
H_2_O + NH_4_NO_3_	3.024	298.15	−24.56	−16.66	26.49
	3.024	303.15	−25.00	−26.58	−5.191
	3.024	308.15	−25.54	−37.13	−37.61
	3.024	313.15	−25.64	−47.59	−70.10
	3.024	318.15	−25.85	−57.91	−100.8
	3.024	323.15	−26.05	−69.75	−135.2
H_2_O + (NH_4_)_2_SO_4_	1.014	298.15	−24.82	−18.15	22.35
	1.014	303.15	−25.15	−21.54	11.94
	1.014	308.15	−25.52	−24.57	3.105
	1.014	313.15	−25.85	−27.35	−4.799
	1.014	318.15	−26.16	−31.79	−17.68
	1.014	323.15	−26.39	−34.03	−23.66

In aq. NH_4_Cl solutions, the Δ*H*^0^_m_ values of the TTAB + PMH system are positive and exhibit a decreasing fashion with enhancing temperature at lower temperatures (298.15, 303.15 and 308.15 K), whereas in the same solvent, the values attain negative magnitude augmented with the increase in temperature (313.15, 318.15 and 323.15 K).

Consequently, the micellization of the current system in aq. NH_4_Cl is endothermic in nature at 298.15, 303.15 and 308.15 K, while the process displays exothermic character at 313.15, 318.15 and 323.15 K. In aq. NH_4_NO_3_ and (NH_4_)_2_SO_4_ media, the Δ*H*^0^_m_ values observed in all investigated temperatures are negative and the −Δ*H*^0^_m_ values experience augmentation with increasing temperature. Therefore, the micellization process in these two salt media is exothermic in nature. The Δ*S*^0^_m_ values are positive in H_2_O + NH_4_Cl medium, while the values are positive and negative at the lesser and higher ranges of working temperature, respectively, in aq. NH_4_NO_3_ and (NH_4_)_2_SO_4_ media. The Δ*H*^0^_m_ and Δ*S*^0^_m_ values indicated the entropy-dominated micellization at a lower temperature in H_2_O + NH_4_Cl medium. The assembly process turns out to be both enthalpy and entropy guided at higher temperatures in aq. NH_4_Cl and lower temperatures in the cases of aq. NH_4_NO_3_ and (NH_4_)_2_SO_4_ media. However, the association is enthalpy controlled in nature in aq. NH_4_NO_3_ and (NH_4_)_2_SO_4_ media at higher temperatures. The Δ*H*^0^_m_ and Δ*S*^0^_m_ values demonstrate that the micellization TTAB + PMH happens through the major contributing forces of exothermic (ion–dipole and dipole–dipole natures) along with hydrophobic interactions.^71,72^ The extent of hydrophobic interaction is significantly larger in the H_2_O + NH_4_Cl medium, while the remarkable contribution of exothermic (ion–dipole along with dipole–dipole types) forces exists among components in aq. NH_4_NO_3_ and (NH_4_)_2_SO_4_ media.

#### 3.1.4. Enthalpy–entropy compensation in the course of micellization

**Fig. 6:**
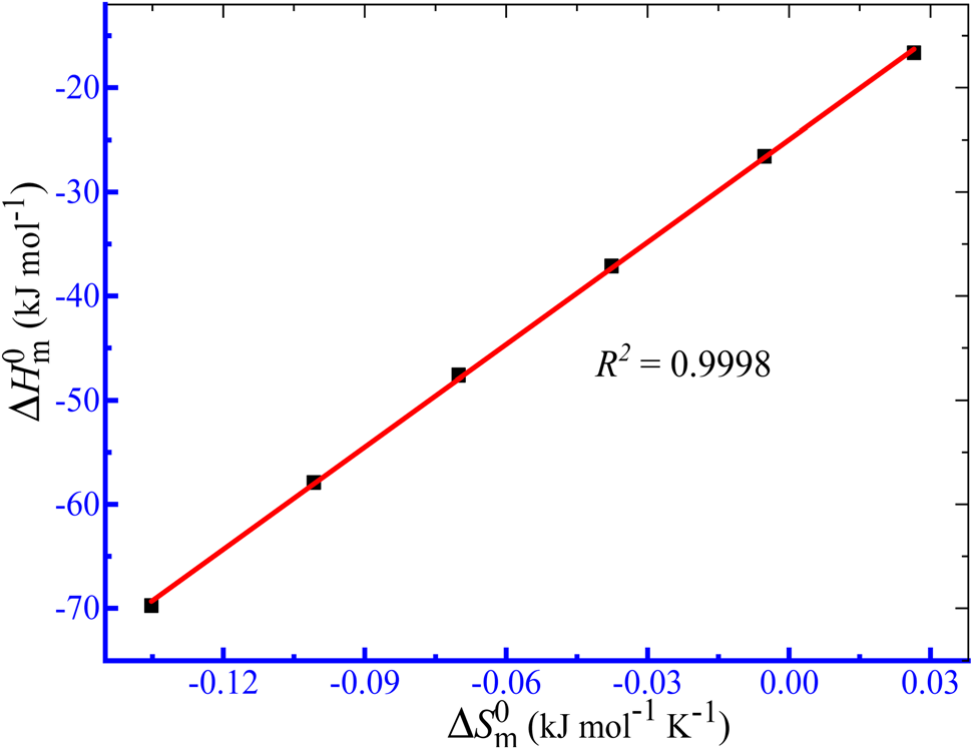
Graph of enthalpy–entropy compensation for the association of the TTAB + PMH (3.009 mmol kg^−1^) mixture in aq. NH_4_NO_3_.

The computed Δ*H*^0^_m_ and Δ*S*^0^_m_ values obtained in the case of the assembly of TTAB + PMH mixtures in aq. ammonium salts were used to inspect the compensation phenomena between the enthalpy and entropy of micellization (Fig. 6) by employing eqn (8):^73,74^8
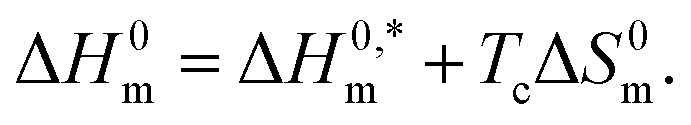


The compensation temperature (*T*_c_), intrinsic enthalpy gain 
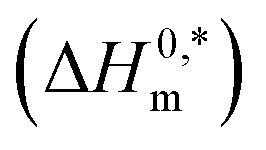
 and *R*^2^ are presented in Table 6. The 
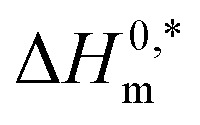
 values demonstrate the solute–solute interaction and disclose the efficiency of the association of hydrophobic moiety in the course of micelle development.^75,76^ The higher negative 
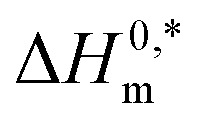
 values refer to the creation of stable drug-induced TTAB micelle. However, the depression of negative 
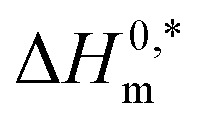
 for TTAB + PMH mixture in other studied media suggests a decrease in stability of the PMH-mediated TTAB micelle. The involvement of water in the solution of variable solutes, including proteins, could be assessed from the numerical values of *T*_c_ values in the range of 270–350 K.^77,78^ The *T*_c_ values of TTAB + PMH solution in the occurrence of ammonium salts are found to be in the range of 293.9–345.4 K. Consequently, the detected *T*_c_ values for the TTAB + PMH mixture in the employed media demonstrate a good comparison with the biological fluids. The observed *T*_c_ values of the current research are also similar to the earlier calculated values for the micelle formation of charged amphiphiles in different additive media.^79–81^

**Table 6:** Values of 
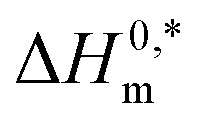
 and *T*_c_ of the micellization of the working mixture in aq. salt media

Medium	*C*_salt_, mmol kg^−1^	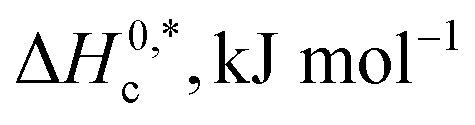	*T*_c_ (K)	*R* ^2^
H_2_O + NH_4_Cl	3.011	−23.893	293.9	0.9996
H_2_O + NH_4_NO_3_	3.024	−24.978	328.3	0.9997
H_2_O + (NH_4_)_2_SO_4_	1.014	−25.734	345.4	0.9997

### 3.2. Clouding phenomena

#### 3.2.1. Impacts of ammonium-based salts on the phase partitioning manner of TX-100 and PMH drug mixture

**Fig. 7:**
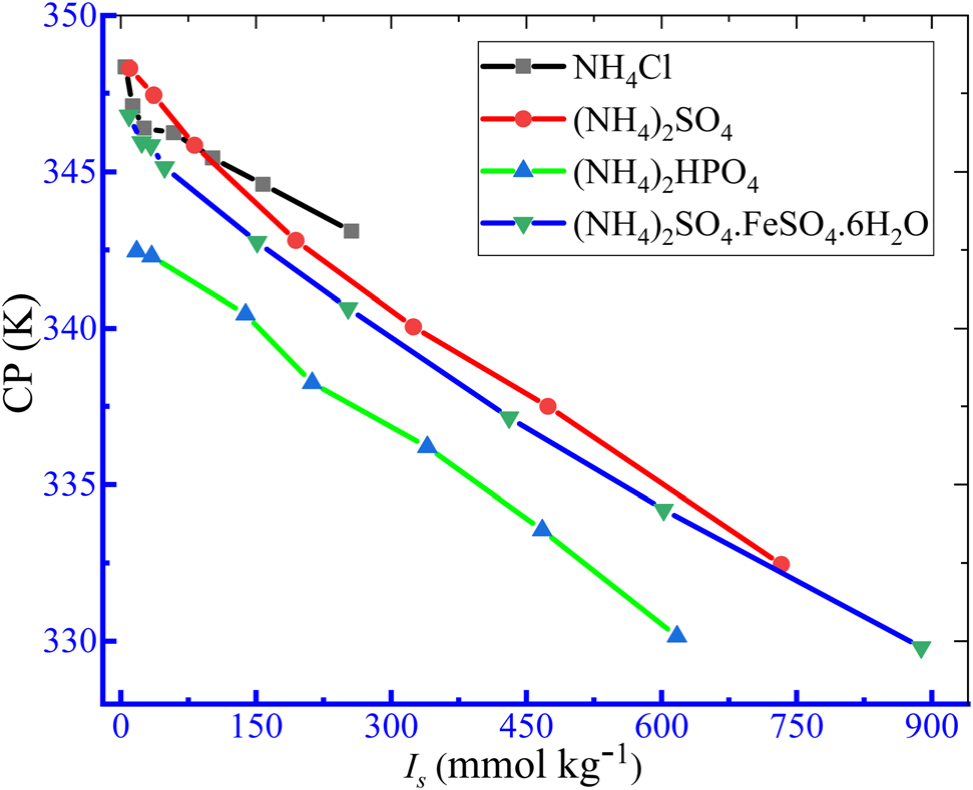
Variation in CP as a function of ionic strength (*I*_s_) of employed ammonium-based salts for the TX-100 + PMH drug mixture in aq. ammonium salt solutions.

The phase partitioning manner of the TX-100 and PMH drug mixture was examined in the manifestation of ammonium salts. In our earlier investigation, the concentration effect of PMH on the cloudy generation of TX-100 in a water medium was inspected taking PMH concentrations of 0.50, 1, 3, 5, 7 and 9 mol kg^−1^, while the CP values of the study system increased with the increase in PMH concentration.^82^ In the current study, a PMH concentration of 1 mmol kg^−1^ was randomly chosen for the clouding phenomenon. The concentration of PMH was not taken similarly to that of micellization because micellization and clouding generation are different physico-chemical processes. In the current examination, four ammonium salts, such as NH_4_Cl, (NH_4_)_2_SO_4_, diammonium hydrogen phosphate – (NH_4_)_2_HPO_4_, and ammonium ferrous sulphate – (NH_4_)_2_SO_4_·FeSO_4_·6H_2_O (AFS), were randomly selected considering two salts (NH_4_Cl and (NH_4_)_2_SO_4_) common to the salts employed in the micellization study. For this purpose, the fixed contents of TX-100 (25 mmol kg^−1^) and PMH drug (1 mmol kg^−1^) were selected. Several concentrations of NH_4_Cl (5.217–256.1 mmol kg^−1^), (NH_4_)_2_SO_4_ (3.334–244.3 mmol kg^−1^), (NH_4_)_2_HPO_4_ (5.875–205.8 mmol kg^−1^), and AFS (1.502–148.0 mmol kg^−1^) were chosen for the investigation, and the respective ionic strengths of the employed ammonium salts are provided in Table 7. In the cases of the micellization study, the ionic strength of salts was employed up to 12 mmol kg^−1^ because of the limitation of the performance of the conductivity meter in salt solutions at high ionic strength. However, in the cloudy creation investigation, the CP values were identified by visual inspection. Additionally, the CP values demonstrate an insignificant change with a slight change in ionic strength; consequently, the study involved a greater extent of salts with high ionic strength (*I*) values. The variation in the CP values of TX-100 and PMH solutions in the incidence of ammonium-based salt media is shown in Fig. 7 and Table 7.

**Table 7:** CP values of TX-100 + PMH (1 mmol kg^−1^) mixed systems at several ionic strengths (*I*_s_) of the employed ammonium salts

Medium	*C*_salts_, mmol kg^−1^	*I*_s_, mmol kg^−1^	*X*_s_ (×10^5^)	CP, K
H_2_O + NH_4_Cl	5.217	5.217	9.396	348.3
	13.23	13.23	23.83	347.1
	25.98	25.98	46.77	346.4
	58.77	58.77	105.7	346.5
	102.1	102.1	183.6	345.5
	157.8	157.8	283.4	344.6
	256.1	256.1	459.2	343.1
H_2_O + (NH_4_)_2_SO_4_	3.334	10.00	6.005	348.3
	12.21	36.62	21.98	347.5
	27.34	82.03	49.22	345.9
	64.77	194.3	116.5	342.8
	108.2	324.7	194.6	340.1
	158.0	474.0	283.8	337.5
	244.3	733.0	438.2	332.5
H_2_O + (NH_4_)_2_HPO_4_	5.875	17.63	10.58	342.5
	11.42	34.25	20.56	342.3
	46.18	138.5	83.11	340.5
	70.87	212.6	127.5	338.3
	113.4	340.3	203.9	336.2
	155.9	467.7	280.0	333.6
	205.8	617.4	369.3	330.2
H_2_O + AFS	1.502	9.009	2.704	346.8
	3.880	23.28	6.988	345.9
	5.584	33.50	10.06	345.9
	8.125	48.75	14.63	345.2
	25.21	151.2	45.38	342.8
	42.04	252.2	75.67	340.7
	71.83	431.0	129.2	337.2
	100.4	602.6	180.6	334.2
	148.0	888.2	265.9	329.8

It was observed that the TX-100 + PMH mixture of CP values decreased as the salt concentrations increased. Above 100 mmol kg^−1^ concentration of employed salts, the CP values of TX-100 + PMH solutions display the following trend: CP (aq. NH_4_Cl) > CP (aq. (NH_4_)_2_SO_4_) > CP (aq. (NH_4_)_2_HPO_4_) > CP (aq. AFS). The “salting out” effect is accountable for depressing the CP values of the study system in ammonium salt solutions. The (NH_4_)_2_SO_4_·FeSO_4_, (NH_4_)_2_HPO_4_ and (NH_4_)_2_SO_4_ release polyvalent anions in H_2_O solution, and this is why they depressed the CP values to a greater extent.^83^ The SO_4_^2−^, HPO_4_^2−^ and Cl^−^ anions, which are documented on the left of the anionic series in the Hofmeister series, are well known for creating the H_2_O structure. Owing to the movement and engagement of H_2_O molecules in the creation of salt anions, the nonionic surfactant TX-100 + PMH system experiences “salting out” phenomena, which reduces the entropies of solution systems (Table 7). Water's dipole has a strong attraction to electrolytes, which salts out the molecules of surfactant from the nearby area and increases inter-micellar interaction, thereby promoting phase separation. The ion–dipole interaction experiences an augmentation with an increase in electrolyte contents; consequently, CP values steadily decline as a function of electrolyte content.^22^

#### 3.2.2. Thermodynamics of phase separation

The thermodynamic parameters of the phase separation were obtained using eqn (9)–(13).^84–86^

**Fig. 8:**
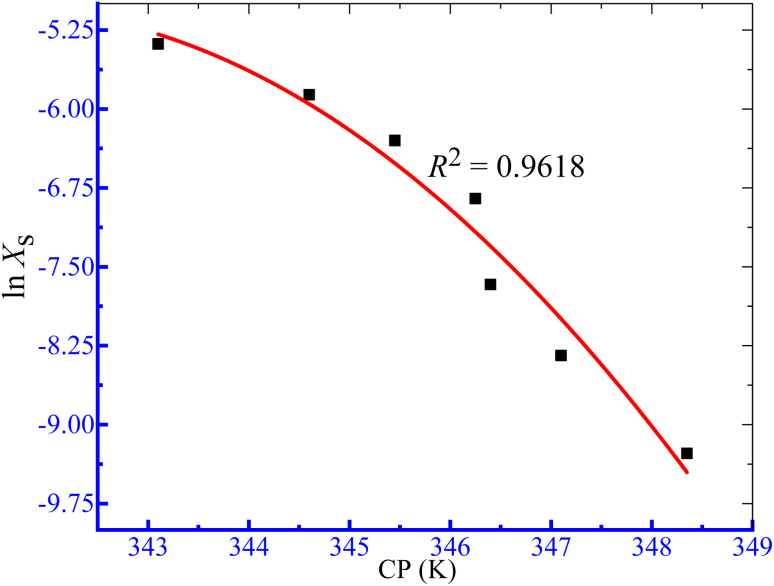
The ln *X*_s_*vs.* CP graph for the clouding of the TX-100 + PMH (1 mmol kg^−1^) mixture an in aq. NH_4_Cl solution.

9 Δ*G*^0^_c_ = −*RT* ln *X*_s_,

10 Δ*H*^0^_c_ = *RT*^2^(∂ ln *X*_s_)/∂*T*,

11 Δ*S*^0^_c_ = (Δ*H*^0^_c_ − Δ*G*^0^_c_)/*T*,

12 ln *X*_s_ = *A* + *BT* + *CT*^2^,

13 Δ*H*^0^_c_ = *RT*^2^[*B* + 2*CT*],

where *X*_s_ is the mole fractional solubility of solutes and *R* and *T* are the gas constant and CP in kelvin, respectively. The variation in CP can be defined as a function of the logarithmic value of the solute's mole fraction (*X*_s_) by eqn (12). The values of regression constants (*A*, *B* and *C*) in eqn (12) were achieved from 2^nd^-order polynomial fitting of ln *X*_s_ against CP plots (Fig. 8), and the values are listed in Table 8. Finally, the Δ*H*^0^_c_ of phase separation was obtained from eqn (13).

**Table 8:** Values of regression constants (*A*, *B* and *C*) for the TX-100 + PMH (1 mmol kg^−1^) mixture in aq. ammonium salt solvents

Medium	*X*_s_ (×10^5^)	*A*	B (K^−1^)	C (K^−2^)	*R* ^2^
H_2_O + NH_4_Cl	9.396–459.2	−10 913	63.886	−0.0935	0.9618
H_2_O + (NH_4_)_2_SO_4_	6.005–438.2	−2152	12.838	−0.0192	0.9999
H_2_O + (NH_4_)_2_HPO_4_	10.58–369.3	−3289.8	19.77	−0.0298	1.000
H_2_O + AFS	2.704–437.4	−1094.8	6.6813	−0.0102	0.9998

The values of Δ*G*^0^_c_, Δ*H*^0^_c_ and Δ*S*^0^_c_ for the TX-100 + PMH mixture in aq. ammonium-salt solvents are presented in Table 9. The positive Δ*G*^0^_c_ values were obtained for the creation of cloudy in the current study system. The +Δ*G*^0^_c_ values experienced a decrease as ammonium-based salt concentration increases, demonstrating that the amount of non-spontaneity of phase separation undergoes a decrease with the increase in salt concentration. The Δ*H*^0^_c_ and Δ*S*^0^_c_ values are negative in almost all the experimented cases, demonstrating that the clouding process is exothermic and enthalpy dominated. The negative entropy values disclosed the highly ordered system in aq. electrolyte media. At the highest salt content, the Δ*H*^0^_c_ and Δ*S*^0^_c_ values become positive. Eminent experts reported the −Δ*H*^0^_c_ and −Δ*S*^0^_c_ values for different surfactant-drug mixed systems in the media of different additives.^87–90^ They also described the interaction forces of H-bonding and dipole–dipole interaction. The Δ*H*^0^_c_ and Δ*S*^0^_c_ values for the working system reveal that the principal interaction forces between components are dipole–dipole (exothermic) and hydrophobic interactions. Additionally, there is a possibility of survival of pi–pi interactions between the drug and surfactant applied for the clouding investigation.

**Table 9:** Thermodynamic parameters of clouding of the TX-100 + PMH (1 mmol kg^−1^) mixture in aq. ammonium salt solvents

Medium	*C*_salts_, mmol kg^−1^	*X*_s_ (×10^5^)	Δ*G*^0^_c_, kJ mol^−1^	Δ*H*^0^_c_ (×10^−1^), kJ mol^−1^	Δ*S*^0^_c_ (×10^−1^), J mol^−1^ K^−1^
H_2_O + NH_4_Cl	5.217	9.396	26.86	−126.7	−371.3
	13.23	23.83	24.07	−102.3	−301.8
	25.98	46.77	22.08	−88.87	−262.9
	58.77	105.7	19.72	−86.00	−254.1
	102.1	183.6	18.09	−70.76	−210.1
	157.8	283.4	16.81	−54.72	−163.7
	256.1	459.2	15.36	−26.79	−82.55
H_2_O + (NH_4_)_2_SO_4_	3.334	6.005	28.15	−54.13	−163.5
	12.21	21.98	24.33	−50.59	−152.6
	27.34	49.22	21.90	−44.02	−133.6
	64.77	116.5	19.25	−31.80	−98.39
	108.2	194.6	17.65	−21.14	−67.37
	158.0	283.8	16.46	−11.55	−39.11
	244.3	438.2	15.01	6.608	15.36
H_2_O + (NH_4_)_2_HPO_4_	5.875	10.58	26.06	−62.42	−189.8
	11.42	20.56	24.16	−61.48	−186.7
	46.18	83.11	20.08	−50.19	−153.3
	70.87	127.5	18.74	−37.07	−115.1
	113.4	203.9	17.32	−25.14	−79.93
	155.9	280.0	16.30	−10.14	−35.28
	205.8	369.3	15.37	8.433	20.89
H_2_O + AFS	1.502	2.704	30.33	−39.34	−122.2
	3.880	6.988	27.52	−37.42	−116.1
	5.584	10.06	26.47	−37.19	−115.2
	8.125	14.63	25.34	−35.63	−110.6
	25.21	45.38	21.94	−30.36	−94.97
	42.04	75.67	20.35	−25.85	−81.87
	71.83	129.2	18.64	−18.58	−60.63
	100.4	180.6	17.55	−12.66	−43.15
	148.0	265.9	16.26	−4.216	−17.71
	243.9	437.4	14.51	11.05	29.89

#### 3.2.3. Enthalpy–entropy compensation of clouding phenomena

**Fig. 9:**
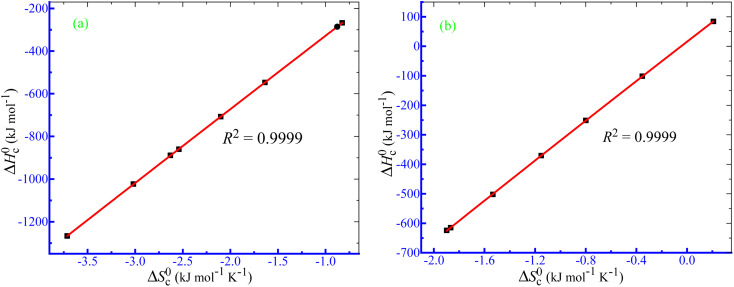
Δ*H*^0^_c_ − Δ*S*^0^_c_ plots for the uncharged amphiphile + used drug mixture in (a) aq. NH_4_Cl and (b) aq. (NH_4_)_2_HPO_4_ media.

In all the circumstances of the current study, excellent linearity between Δ*H*^0^_c_ and Δ*S*^0^_c_ was obtained (Fig. 9). The respective compensation parameters were determined using eqn (14):^91,92^14
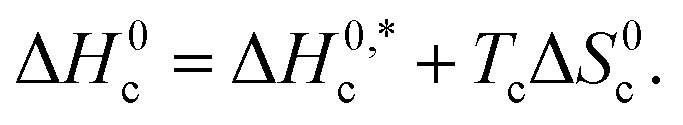


The 
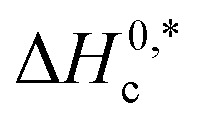
 and *T*_c_ values for the current system in aq. ammonium salt environment are summarized in Table 10. From 
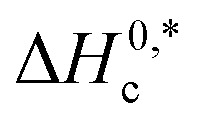
 values, one may estimate the existing solute–solute interaction, and from *T*_c_ values, one can analyze the solute–solvent interaction.^93–95^ All the analyzed examples had positive-estimated 
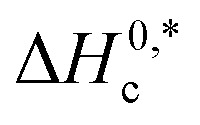
 values, indicating that the system becomes unstable at the stage of cloudy development. The *T*_c_ values for the TX-100 + PMH mixture fall between 334.1 and 345.6 K. The investigational outcomes show a decent resemblance with the stated *T*_c_ value for biological fluids and tiny solute solutions.^78^

**Table 10:** Values of 
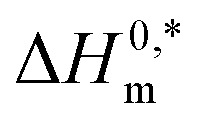
 and *T*_c_ of the clouding process of the TX-100 + PMH (1 mmol kg^−1^) mixture in aq. ammonium salt media

Medium	*C*_salt_, mmol kg^−1^	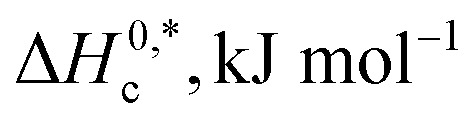	*T*_c_ (K)	*R* ^2^
H_2_O + NH_4_Cl	5.217–256.1	18.27	345.6	0.9999
H_2_O + (NH_4_)_2_SO_4_	3.334–244.3	16.19	341.0	0.9999
H_2_O + (NH_4_)_2_HPO_4_	5.875–205.8	16.23	337.3	0.9999
H_2_O + AFS	1.502–243.9	14.56	334.1	0.9998

## 4. Conclusions

This study demonstrates the plausible interactions of TTAB/TX-100 with PMH in aq. ammonium salts media at variable temperatures (ranging from 298.15 K to 323.15 K) and the compositions of salts. The experiments are conducted through conductivity and cloud point measurement tools. A single CMC is detected for the association of TTAB and PMH solution in the presence of different salts. The micelle formation acquired depends on the nature of the salts, their concentration, and the temperature change. The micellization is attained to be augmented in the presence of salts while delaying micelle creation with the increase in the experimental temperature. The CP values of TX-100 + PMH drug decrease in the occurrence of salt, demonstrating the decrease in the solubility of the study system. The magnitudes of free energy of micellization (Δ*G*^0^_m_) and clouding (Δ*G*^0^_c_) in the surfactant + PMH mixtures were perceived to be negative and positive, which demonstrated that the processes are spontaneous and non-spontaneous, respectively. In the aq. ammonium salt systems, in most cases, the micellization/clouding of TTAB + PMH mixtures was exothermic, as shown by the appearance of negative Δ*H*^0^_m_/Δ*H*^0^_c_ with exceptions in aq. ammonium chloride solution at lower temperatures (the micellization is endothermic in behavior). In all tested media, the computed Δ*S*^0^_m_/Δ*S*^0^_c_ values for the TTAB + PMH mixtures were also observed to be positive/negative in the respective processes. The Δ*H*^0^_m_/Δ*H*^0^_c_ and Δ*S*^0^_m_/Δ*S*^0^_c_ values for the TTAB/TX-100 and PMH mixtures in aq. ammonium salt solutions clearly imply that the interactions between surfactants and PMH molecules are most likely to be hydrophobic, ion–dipole/dipole–dipole, and electrostatic forces. There is also a probability of the existence of pi–pi interactions between TX-100 and PMH drug. The important discoveries acquired from this study could be helpful in the industrial formulation of amphiphiles in aq. ammonium salt systems. The solubility nature of the study system in ammonium salts will also impart to the storage and extraction processes. In the future, this work can be expanded to include a wide range of additional potential solvent mixtures with a greater focus on system characterization by utilizing various advanced methods, such as TEM, SEM, ITC, and molecular dynamics simulations.

## Conflicts of interest

There are no conflicts of interest to declare.
